# Sex differences in the genetic architecture of clinical quantitative traits in the electronic health record

**DOI:** 10.64898/2026.09.02.26362069

**Published:** 2026-09-07

**Authors:** Freida Blostein, Banabithi Bose, Andrew Hill, Ky’Era Actkins, Allison Lake, Naomi Freilich, Peter Straub, Maria Niarchou, Barbara E. Stranger, Lea K. Davis

## Abstract

Sex differences exist in complex diseases, but the genetic architecture of sex differences in quantitative clinical traits is understudied. Here, we performed sex-stratified genome-wide association studies of 508 clinical traits from electronic health records (EHRs) in 64,956 European-ancestry individuals in the Vanderbilt biobank, BioVU, with replication in the UK Biobank and Colorado Center for Personalized Medicine biobank. Most trait distributions differed significantly by sex. We identified a female-specific locus for erythrocyte distribution width at *PIEZO1* and sex-differentiated effect magnitudes at *APOE* (LDL cholesterol) and *SLC2AS* (uric acid) that replicated across biobanks, varied with age, and partially mediated associations with heart disease and gout. Opposite-direction sex effects were less replicable. Ascertainment bias could be addressed using inverse-probability weighting. Sex-stratified colocalization with eǪTLs nominated additional candidate genes. Differential effect magnitude, not opposing effects, predominate in sex- specific genetic architecture, requiring large sample sizes for discovery that can be achieved using EHR data.

## Full Text Availability

The license terms selected by the author(s) for this preprint version do not permit archiving in PMC. The full text is available from the preprint server.

